# Social jetlag is associated with adverse cardiometabolic latent traits in early adolescence: an observational study

**DOI:** 10.3389/fendo.2023.1085302

**Published:** 2023-07-04

**Authors:** Sabine Pompeia, Sareh Panjeh, Fernando Mazzili Louzada, Vania D’Almeida, Debora Cristina Hipolide, Hugo Cogo-Moreira

**Affiliations:** ^1^ Departamento de Psicobiologia, Universidade Federal de São Paulo, São Paulo, Brazil; ^2^ Departamento de Psiquiatria, Universidade Federal de São Paulo, São Paulo, Brazil; ^3^ Departamento de Fisiologia, Universidade Federal do Paraná, Curitiba, Brazil; ^4^ Department of Education, ICT and Learning, Østfold University College, Halden, Norway

**Keywords:** adolescence, social jetlag, sleep, metabolic syndrome, cardiometabolism

## Abstract

**Introduction:**

Adolescence is marked by physiological and social changes, such as puberty, increased responsibilities and earlier school start times. This often leads to insufficient sleep on school nights and the need to compensate for lost sleep on weekends, causing a misalignment between biological and social times, which has been termed social jetlag (SJL). SJL triggers stress responses and is associated with several negative health outcomes, including higher cardiometabolic risk in adults. In adolescence, however, SJL has only been consistently related to increases in adiposity but its association with other cardiometabolic indicators are unclear.

**Method:**

In a sample of 278 healthy early adolescents (9-15 years of age; 168 girls) we investigated: 1) whether self-reported SJL is associated (using path analyses) with a cardiometabolic status latent factor obtained by testing the best fitting model *via* confirmatory factor analyses from an initial set of eight indicators [body mass index (BMI), waist/height ratio, triglyceride concentration, diastolic and systolic blood pressure, glycated hemoglobin, total cholesterol/high-density lipoprotein ratio (chol/HDL), and % body fat]; and 2) whether age and/or pubertal status influence the association between SJL and cardiometabolic status.

**Result:**

We found that, for girls, higher SJL was associated with more adverse cardiometabolic latent scores (the shared variance of BMI, waist/height ratio, chol/HDL and systolic blood pressure, which had acceptable model fit indices). However, the role of age and pubertal status in this association was unclear for both sexes.

**Discussion:**

SJL was associated with adverse cardiometabolic latent traits beyond increases in adiposity in this observational study in early female adolescents. Because disruptions of circadian rhythms are believed to lead to dysregulated energy homeostasis and not vice-versa, our findings highlight the need for sleep interventions in adolescence to help reduce the global burden of cardiometabolic ill health, especially in girls.

## 1 Introduction

Sleep patterns change in adolescence due to various social factors, such as more freedom to choose bedtimes, greater use of electronic devices in bed and having more responsibilities and tasks to complete, all of which can lead to later bedtimes. Adolescent sleep patterns also changes due to physiological factors, with their sleep phase being delayed and their increasing capacity to resist the accumulation of homeostatic sleep pressure over the waking day, making them prone to go to bed later than during childhood; however, the capacity to dissipate sleep pressure remains immature throughout most of adolescence, so they still need to sleep longer than adults (1). The effect of these social and biological factors, combined with early school start times, means that adolescents do not usually get all the sleep they need. Hence, adolescents worldwide often try to compensate for this sleep dept by sleeping longer on weekends (2–5). This weekly change in sleep patterns is akin to travelling from one time zone to another at weekly intervals, creating what is termed “social jetlag” (SJL): a “misalignment of biological and social time” that can be measured as the absolute difference in the midpoints of the sleep period on school nights and on days off school (6).

SJL reaches a peak in adolescence (4) and exerts considerable negative effects on physical and mental health (7), including on behavioral/mood changes, as well as affecting academic performance and increasing proneness to accidents (1, 8). Of interest here is the relationship of SJL with adolescent cardiometabolic health, a cluster of factors that include elevated adiposity (a high body mass index [BMI], percentage of body fat, and waist circumference), glucose intolerance, insulin resistance, increased triglyceride concentrations, reduced high-density lipoprotein cholesterol (HDL) and hypertension (see 9, 10). These relations are believed to be driven in part by an increase in stress brought about by short and/or irregular sleep patterns (11), which are associated with blood glucose, insulin concentrations and blood pressure, chronically culminating in cardiometabolic dysregulation (11, 12), even in young adolescents (13) and healthy adults (14). Indeed, the extent to which chronic stress effects (allostatic load) and cardiometabolic risk are differentiable is still a matter of debate (15, 16).

The relation of SJL and cardiometabolic risk is believed to be greater than that of reduced sleep duration/quality, and has been found to be associated with higher measures of adiposity (e.g., BMI) in adults (3) and pre-adolescents (17, 18). In adolescents, SJL (whether adjusted or not for age and sex) is associated with increased BMI (8, 19–23), and other adiposity measures such as waist/height ratio (20), fat mass (21, 23) and waist circumference (21) (for different findings, see 24–26).

Although it seems clear that there is a positive association between SJL and adiposity irrespective of peoples’ age, SJL is also related to components of cardiometabolic risk and adverse endocrine profiles other than adiposity in pediatric (27) and adult populations (14, 28–30). However, this association is less clear in adolescence, although in a variety of other sleep disturbances (e.g., short sleep duration and bad quality sleep) there appear to be a number of factors, other than high adiposity, that can increase the odds of presenting worse cardiometabolic profiles in adolescents, such as higher diastolic (31) or systolic blood pressure (32), hypercortisolemia (33), lower HDL cholesterol (34), elevated fasting glucose and higher triglyceride concentrations (34).

Two studies in early adolescents (seemingly with the same/overlapping large sample: 21, 35) explored this issue at a deeper level using a comprehensive cardiometabolic score that combined various measures of cardiometabolic risk (mean sex-specific z scores for waist circumference, systolic blood pressure, HDL, triglycerides and insulin resistance) and controlled for obesity-related behaviors such as physical activity. Although the first of these studies (35) reported that shorter sleep duration and worse sleep efficiency related to this general cardiometabolic risk composite score, corroborating the abovementioned findings for individual cardiometabolic markers, the later study (21) did not find this combined score to be related to SJL *per se* (21), despite having seemly used the same sample. Hence, it is still unclear whether SJL is directly associated with cardiometabolic dysregulation in adolescence, beyond increases in adiposity.

Here, our objective was to study the association between SJL and cardiometabolic risk determined using a novel approach (latent factor; detailed below) in adolescence, an age in which there is a peak in SJL (4) and the prevalence of cardiometabolic problems has risen worldwide in recent years (36). We focused on the first half of adolescence (below the age of 16 years), the time of life in which the pubertal transition takes place (37, 38) and used a non-clinical sample. The reason for this was that if an association between SJL and cardiometabolic health is confirmed, this would be an ideal age to explore the implementation of sleep-related interventions aimed at reducing later development of cardiometabolic risk because: 1) the rate of cardiometabolic ill health doubles in older adolescents compared with younger individuals, possibly due to pubertal-related mechanism (39); and 2) overweight/obese children who grow up to be adults of a healthy weight are less prone to develop cardiometabolic problems later in life than people who have unhealthy weight from childhood to adulthood (9).

We also studied sex effects on the association between SJL and cardiometabolic health because during early adolescence body composition, growth in height and metabolism change differently in each sex (40–43), with boys acquiring more lean and skeletal mass as they develop, while girls accumulate more fat mass, although the mechanisms and directionality of these associations are not yet fully understood (43). In this respect, two studies (18, 21) found a relation between SJL and adiposity measures only in girls. Additionally, among girls entering adolescence who presented SJL, the odds of being overweight or obese were much higher (18). Adolescent girls also seem to exhibit higher SJL than boys (8, 18, 41, 43–45), although this is not confirmed in all studies (5). Thus, it is important to consider sex differences when studying the association between SJL and cardiometabolic status in adolescents to identify individuals who are at a higher risk of developing cardiometabolic problems, especially because a prospective study showed a higher negative impact of SJL on age- and sex-standardized BMI and fat mass between the ages of 12 and 15 years (23).

Apart from considering the possible sex- and age-specific associations of SJL and cardiometabolic indicators, we also addressed some statistical issues that have limited the findings in this field. The first is the prevalent use of standardized scores of variables such as BMI (corrected for age and sometimes for sex) when no such correction is applied in respect of SJL or other cardiometabolic measures, confounding results. Moreover, many previous studies grouped individuals in different BMI and/or SJL categories using different arbitrary cut-off criteria instead of determining linear effects, which makes it difficult to compare the results among studies. Also, when more than one indicator of cardiometabolic heath was used, most studies ran statistical models for each indicator separately, which increased the risk of Type I errors (false positive results), often disregarding the fact that such variables are probably intercorrelated, which can also inflate the chance of obtaining multiple significant effects. This can explain why the non-adiposity cardiometabolic factors associated with sleep disturbances (other than SJL) in adolescents are inconsistent among studies (e.g., 31–34).

We therefore opted to use continuous SJL scores and a single comprehensive continuous, non-age and sex-adjusted measure of cardiometabolic health to avoid arbitrary cut-off scores and Type I errors. To determine the cardiometabolic score we employed a data-driven approach to form a latent variable considering various cardiometabolic markers that produced a good fitting model using Confirmatory Factor Analysis (CFA: see details in the Methods section). Latent factors represent the shared variance of components or parameters (cardiometabolic variables or indicators) presumed to underly a theoretical construct (in our case, cardiometabolic health) whereas the variance that is unique to each indicator, that is, that does not share variance with the other indicators (errors or residuals) is partialled out, enhancing the validity of the analyses (46). The method employed here avoids the use of latent variable binary splits based on arbitrarily defined cut-offs because continuous values in respect of the cardiometabolic parameters can be used. Additionally, the contribution (or weight) of each indicator on the latent factor is expressed as its loading on this latent variable, allowing researchers to determine which ones exert greater effects; these indicators can also vary across studies, as long as they reflect the same construct (46). Accordingly, a number of studies have shown that using a latent cardiometabolic status factor has many advantages in comparison to the use of metabolic syndrome indexes or of various indicators separately (47, 48). This is considered a superior method compared to others commonly used ones such as the metabolic syndrome index, which uses arbitrary cut-off scores, regards all cardiometabolic measures as having equal weight in the final score and involves inconsistent adjustments for sex, age and/or pubertal status in respect of some but not all variables^1^.

The main objective of the present study was, therefore, to determine, in sex-specific statistical models, whether self-reported SJL (as a continuous variable) is associated with worse cardiometabolic profiles (beyond increases in measures of adiposity). This was measured as a latent factor (shared variance among various continuous cardiometabolic biomarkers) in the first half of adolescence, which encompasses the pubertal transition. We hypothesized that we would replicate the positive association of SJL and cardiometabolic ill health found when using individual adiposity markers as described above, particularly in girls because: 1) sexual maturation has larger effects on metabolism in this sex; and 2) girls have been found to exhibit higher SJL (8, 18, 41, 43–45).

A second objective was to explore if the association between SJL and cardiometabolism is altered when the statistical models are controlled for development considering both chronological age and pubertal status. Prior studies determined the association of sleep problems and cardiometabolic profiles averaged across adolescents of different ages and sexual maturation stages, the latter of which varies widely among individual in terms of pubertal timing (the age when puberty begins) and its rate of progression (37, 38). The fact that stages of pubertal development are disregarded in this literature is surprising given that increases in age and higher sexual maturity are associated with physiological changes that can be partly responsible for both increases in SJL across adolescence (5) and also worse cardiometabolic profiles [increases in insulin resistance, obesity, and lower levels of HDL (39)]. Hence, as adolescents become older and more sexually mature they would be expected to exhibit greater SJL and cardiometabolic risk. Therefore, it could very well be that the association of SJL and cardiometabolic status differs at distinct developmental stages. In other words, adolescents’ age and/or pubertal status could affect (increase or decrease) this association, especially in girls as mentioned above.

## 2 Materials and methods

### 2.1 Participants

We used a non-clinical convenience sample (N=278; 168 girls) of typically developing native Portuguese-speakers from the city of São Paulo, Brazil, aged 9 to 15 years old, an age span that includes the full range of pubertal development stages (37, 38). The exclusion criteria (based on reports given by guardians) were: 1) the use of chronic medication, to rule out the presence of clinical disorders and/or use of drugs that could impact development, sleep and/or cardiometabolic profiles; 2) having been held back at school for a year or more or being a student with special needs, which would be indicative of the presence of possible clinical problems. See below for information on sample size estimation.

### 2.2 Procedure

This cross-sectional observational study received ethical approval from the Ethics Committee of the Universidade Federal de São Paulo (CAAE 56284216.7.0000.5505). After obtaining permission from a number of schools in various areas of the city, arrangements were made with the staff to invite potential participants and their families to take part in the study. Those who expressed an interest in being involved in the study took home written instructions, consent forms and questionnaires (regarding demographics and medical history, among other factors) to be completed by one of their guardians. Participants were only included if they met the eligibility criteria, assented in taking part and returned signed informed consent forms from their guardians. Participants were tested at their school in two sessions. The first was an individual session in which they completed questionnaires (including reports on sleep patterns and on self-rated pubertal status). The second was a group session, during which a trained team obtained anthropometric measurements and capillary blood from the participants for the determination of their lipid profiles and glycated hemoglobin levels. We could not fully control for circadian influences regarding cardiometabolic biomarkers because Brazilian students study either in the morning or in the afternoon, as the schools use a “shift” system. Nonetheless, to minimize these circadian effects, we collected the biological data towards the end of the morning and the beginning of the afternoon.

After data collection, the participants were awarded a “Science Partner” certificate and reimbursed for their commuting costs on the days of the experiment. The families received an individualized report of the findings. Referrals were provided when results indicated cardiometabolic problems in compliance with local clinical standards. Other information was obtained from the participants and their guardians but was not used in the current study.

### 2.3 Measures

#### 2.3.1 Developmental measures

Chronological age: participants reported their date of birth, which was used to determine their age in months.

The Pubertal Development Scale (PDS) (56), adapted for local use (57). This scale was completed by the participants to assess the level of development of the following secondary sexual characteristics: growth spurt, growth of body hair and skin changes for participants of both sexes, voice changes and facial hair growth for boys, and breast growth for girls, all measured using a 4-point scales ranging from 1 “not yet started” to 4 “seems complete” (with 0 “I don’t know”, coded as missing). For girls, there was an additional question with a dichotomous answer, about the occurrence of menarche (score 1 = no; 4 = yes). The scores were the mean ratings of all five sex-specific questions.

#### 2.3.2 Social jetlag

Participants gave information on the clock time when they usually went to sleep (sleep onset) and woke up (sleep offset) on weekdays and on days off, that is, days in which they did not have to wake early to attend church or other similar activities (hereafter named weekends). SJL was measured as the difference, in hours and minutes (transformed to decimal numbers), between the mid-sleep point on school nights and the mid-sleep point on days off, non-corrected (6). For example, if a participant reported sleep onset on weekdays at 00:00h and sleep offset at 06:00h, the mid-point was 03:00h. If the same individual reported sleep onset was 01:00h and the sleep offset at 11:00h on weekends, the midpoint was 06:00h. In this case, SJL was the difference between 03:00h and 06:00, that is, three hours. Hence, our SJL metric was a raw, continuous bidirectional value, with positive values indicating a later midsleep point on days off than on school nights, and negative values indicating the inverse.

#### 2.3.3 Cardiometabolic markers

- Anthropometric measures were obtained using calibrated instruments following a standardized protocol described by Frisancho (58) using the means of two measurements. Weight (kg) was determined with an OMRON HBF-514C Body Control scale after participants had removed their coats and shoes. Height was obtained using a portable stadiometer. BMI was calculated using the formula BMI = kg/m2. The percentage of body fat was estimated with an OMRON HBF-514C Body Control scale by bioelectrical impedance across hands and feet, considering the participants' age, gender and height following the instructions in the equipment manual. Waist circumference (cm) was measured with a tape over the navel, mid-way between the lowest rib and the iliac crest, and was divided by height (cm) to produce the waist/height ratio.

- Diastolic and systolic blood pressure were measured twice in seated participants using an Automatic Arm Blood Pressure Monitor model HEM-7122 ^®^ that uses the oscillometric method of blood pressure measurement. The mean values were used in the analyses.

- Lipid profiles (total cholesterol and fractions, triglycerides) were obtained with a point-of-care Cardiochek PA analyzer^®^ (Polymer Technology Inc, Newark, DE, USA) [for reliability, see Ferreira et al. (59)]. Capillary blood (40 μl for each equipment) was collected in a tube after puncturing a finger with a lancet, following the recommendations of Krleza et al. (60). The quantitative results for the lipid profile given by the Pts panel test strips vary from 100-400 mg/dl for total cholesterol, 15-100 mg/dl for HDL fraction and 50-500 for triglycerides, which comprised the measurements in our sample. - In the very rare cases in which lower levels were found, the values were substituted for the minimum values (respectively 100, 15 and 50) in the analyses.

- Glycated hemoglobin (HbA1c) was obtained with point-of-care A1CNow SystemPTS Diagnostic^®^ equipment (for reliability, see 61), from capillary blood as for lipid profile.

### 2.4 Statistical analyses

There was no imputation of missing data in any of the analyses. Descriptive statistics for all variables were determined using the statistics package SPSS version 26.0 (62). The same software was used to compare SJL between sexes (Mann-Whitney-U-Test was conducted due to the lack of the normality assumptions for t-test), and we also provided effect sizes.

**Figure 1:**
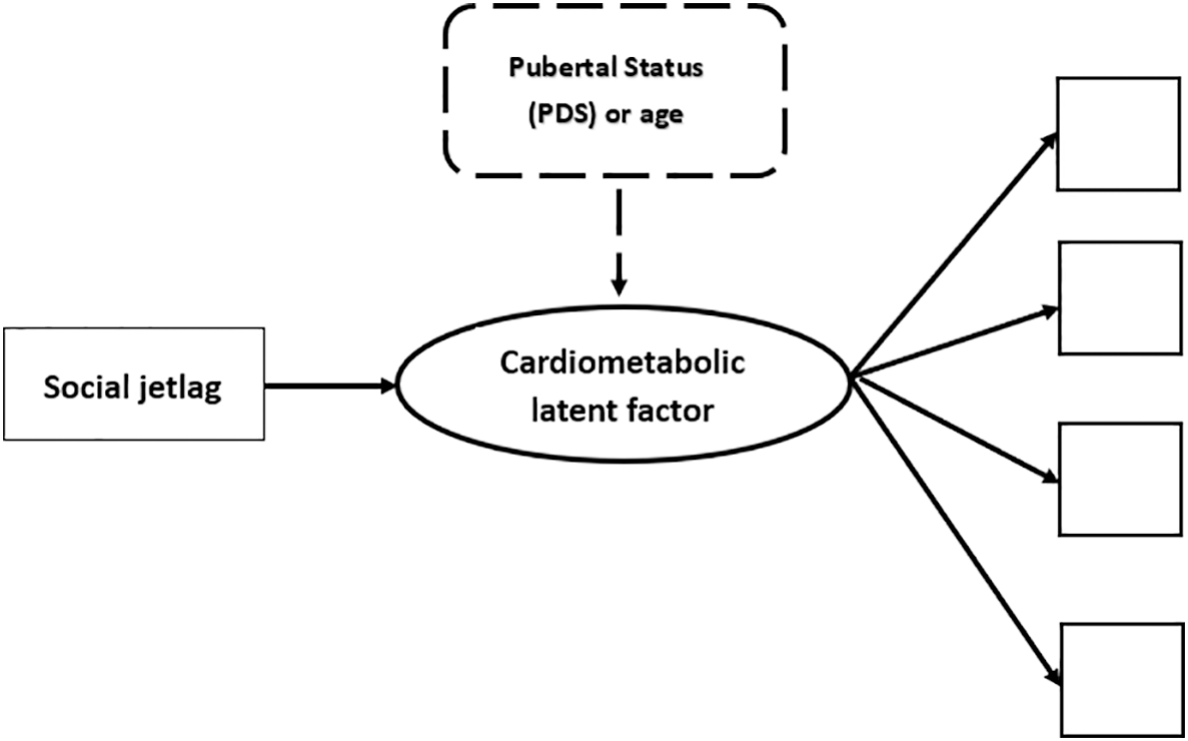
Illustration of the tested theoretical models. NB. In thick lines there is a representation of the cardiometabolic latent factor (circle), that is, the common variance between observed scores/values of various cardiometabolic indicators, each of which is represented as a square. The first tested path analyses explored the association of social jetlag with the cardiometabolic latent factor and this is represented by the continuous thin arrow linking these variables. The subsequent tested step in the path analyses is illustrated in dashed lines. This was done by adding a control of developmental markers on the cardiometabolic latent outcome. Two developmental controls were used in different models: 1) scores in the Pubertal Development Scale (PDS); or 2) age in months. All analyses were conducted separately for girls and boys.

To allow comparisons with prior literature and to decide which cardiometabolic markers would compose the tested cardiometabolic latent factor (see below) we determined the Pearson inter-correlations between variables using the same statistical package. The other analyses (CFA and path modeling) were conducted using Mplus version 8.2 computer package (63). **Figure 1** illustrates the tested path models.

#### 2.4.1 Determination of sample size

Sample size was determined based on rules of thumb, i.e., a minimum of 10 participant per indicator (64). Our models with the most indicators were the path analyses, which are an extension of multiple regressions that allows for the analysis of complex models, such as those that use latent factors, as in the present case. These analyses potentially included a maximum of ten variables (SJL, eight cardiometabolic indicators to form the cardiometabolic latent factor and one mediator or moderator). Hence, a minimum sample of around 100 participants would be required per sex if all cardiometabolic indicators were found to compose the latent factor. To increase the chances of having models with acceptable fit, the CFA and path analyses were run using Bayesian (65) instead of frequentist statistics, which are more appropriate for smaller samples (66). This is detailed below.

#### 2.4.2 Cardiometabolic latent factor

Because there is no consensus of which indicators should be used to build a cardiometabolic latent score, we followed the series of steps described in detail in **Supplementary File 1** to find the best indicators. To choose these biomarkers we started from an initial set of eight variables regarded as measures of adiposity and cardiometabolic risk (47, 54, 67, 68) similar to those used in some studies that proposed cardiometabolic latent factors in adolescents (10, 49, 69), namely: glycated hemoglobin, which indicates the control of body glucose over the last few months; lipid profile, including triglyceride concentrations, the cholesterol ratio (total cholesterol divided by HDL, which reflects the *balance* of atherogenic cholesterols, the major determinant of cardiovascular risk) (70), anthropometric/adiposity measurements (BMI, waist/height ratio and percentage of body fat, which are measures of adiposity) and systolic and diastolic blood pressure, which are cardiovascular indicators.

Briefly, because many studies have found that the cardiometabolic profile composes a single latent factor (e.g. 10, 49, 69, 71, 72), we aimed to replicate this single factor with our data and did not explore alternative factor configurations. To build our model we followed a series of steps that are detailed in the **Supplementary File 1** in the interest of transparency. We began by examining the Pearson intercorrelation of observed variables in boys and girls separately because growth, cardiometabolic and body composition change differently in each sex in adolescence (40–43). We excluded indicators that did not intercorrelate with at least one other variable with values higher than [ ± ]0.3 (low effect size) (73) and also those that could present collinearity. With the remaining indicators we used Mplus to conduct CFA to determine the adequacy of a single latent factor in explaining the covariance among the observed cardiometabolic indicators (74) for both sexes separately. If the model did not converge, we removed indicators with lower factor loadings while maintaining indicators that are biomarkers of cardio metabolism until a convergent model with the best fit was obtained.

Given the impossibility of determining sample size based on the lack of prior studies using similar models, we ran all analyses using Bayes estimators (66). Proportional scale reduction (PSR) was used to estimate convergence. Bayesian analyses apply Markov chain Monte Carlo algorithms based on Gibbs sampler (75) to iteratively obtain an approximation to the posterior distributions of the parameters. In this approach, the variation of the parameters estimated in each iteration (called a chain) is compared. The essential criterion of PSR requires the between-chain variation to be smaller than the total of between- and within-chain variations. The models were run first with the default iteration of Mplus and two chains until the chain goal reached a PSR value close to 1.0 for all iterations after burn-in (the first half of the iterations) (65). Posterior predictive *p* values (PPP) were applied to evaluate the structural model for misspecifications. In this approach, estimated posterior distribution is used to evaluate how well the posterior distribution and the model fits the data (76). A PPP close to 0 or 1 indicates poor fit, while intermediate values suggest acceptable fit; the closer to 0.5, the better. PPP reflects the proportion of times that the observed data are more probable than the generated data (i.e., the proportion of times observed data have a smaller χ2 than the generated data). Thus, PPP values close to 0.50 indicate that, on average, the observed data are just as probable as the generated data, implying a good model fit. However, the smaller the PPP values the poorer the fit (see http://www.statmodel.com/). The 95% confidence interval (CI) for the difference between the observed and the replicated chi-square value should range from a negative to the positive value, centered on zero (65, 75). Since similar studies that evaluated the same hypothesis as tested here were not found and because in small to medium samples informative priors can influence results (see http://www.statmodel.com/), the non-informative prior was used: Mplus applies different default non-informative priors for different parameter of models (i.e., factor loading, intercepts, regression coefficients), including inverse-gamma, normal, and inverse-wishart priors (i.e., IG(-1, 0), N(0,∞), IW(0,-3) (65, 76).

#### 2.4.3 Path analyses to determine the association of SJL with the cardiometabolic latent factor and the influence of age/pubertal status on this association

Once acceptably fitting sex-specific cardiometabolic latent factors were obtained, the next step was to determine whether SJL associated with the cardiometabolic latent factors for each sex separately (see **Figure 1**). We then investigated (full path model) whether the effect of SJL on the cardiometabolic latent factor changed when developmental markers were used as controls on the outcome (pubertal status assessed with the PDS scores and, in comparable models, age in months), separately for each sex. The adequacy of these models was determined using the same model fit indices described above using Mplus.

### 2.5 *Post hoc* exploratory analyses of the association of SJL and cardiometabolic indicators and the influence of age/pubertal status on this association

Because we failed to find good fitting path models when we controlled for age and PDS scores in the path analyses (see Results), we conducted other, exploratory *post hoc* analyses using SPSS v.26.0 to determine whether these developmental markers were associated with SJL and cardiometabolic indicators that were found to form the cardiometabolic latent factor. The first exploratory analysis were Pearson correlation matrices controlled for multiple comparisons to describe linear inter-associations between the individual cardiometabolic status indicators, SJL, and developmental markers (age and PDS), by sex.

The next exploratory analyses were multivariate analyses of covariance (MANCOVA) in which all four metabolic indicators used to build the latent factors (BMI, waist/height ratio, systolic blood pressure, and total cholesterol/HDL) were entered as multivariate dependent variables, and SJL and PDS (or age in separate models) as continuous predictors, including the correlation of SJL and the developmental measures (PDS or age), per sex. The level of significance was 5%. When Pillai’s Trace values were significant, follow-up univariate analyses for each cardiometabolic indicator were carried out. If interactions of SJL and developmental markers was significant, Johnson-Neyman plots (77) were use to interpret effects. Effect sizes were determined with partial eta squared (η_p_^2^): values between 0.059 and 0.138 are considered medium effect sizes and those higher than 0.138, large effect sizes (73).

## 3 Results

The databank is available at https://osf.io/5mv6w. All Mplus syntaxes can be found in **Supplementary File 2** at the same OSF depository.

### 3.1 Characteristics of the sample

All descriptive statistics are shown in **Table 1**, including sample size per variable as we did not impute missing data. The result of Mann-Whitney-U-Test showed that there is no difference between girls and boys in terms of SJL (Z= -1.037; p=0.300; R^2 =^ 0.004).

**Table 1:** Descriptive statistics of assessed variables, per sex.

VARIABLES	GIRLS					BOYS				
	N	Minimum	Maximum	Mean	SD	N	Minimum	Maximum	Mean	SD
DEVELOPMENTAL										
Age (months)	168	111	193	159.69	20.42	110	114	197	154.18	22.02
Pubertal Develop. Scale (score)	168	1.20	4.00	02.81	00.70	109	01.00	04.00	02.22	00.64
CARDIOMETABOLIC										
Body Mass Index (BMI; kg/m^2^)	147	14.50	37.34	21.20	04.11	94	13.71	32.80	21.03	04.23
Body fat (%)	146	5.15	50.25	29.11	09.65	69	05.55	38.55	19.03	09.34
Waist/height (ratio)	146	0.32	0.61	0.46	00.05	94	00.38	00.65	0.48	00.07
Triglycerides (mg/dl)	143	49.00	365.00	97.92	50.41	93	49.00	278.0	104.5	49.10
Total cholesterol/HDL (ratio)	139	01.75	05.73	02.95	00.74	92	01.44	06.30	03.07	00.85
Systolic blood pressure (mmHg)	148	82.50	131.50	104.2	10.20	95	82.00	135.5	108.0	11.20
Diastolic blood pressure (mmHg)	148	53.0	99.50	70.00	07.70	95	56.00	90.50	70.20	07.33
Glycated Hemoglobin (%)	142	04.00	07.50	05.27	00.46	90	04.10	06.90	05.30	00.45
SLEEP										
Sleep onset weekday (h)	163	20.00	02.00	22.70	01.46	109	19.00	02.50	22.57	01.54
Sleep offset weekday (h)	166	04.00	12.00	07.67	02.02	108	04.00	11.50	07.53	01.94
Sleep onset weekend (h)	164	21.00	05.00	00.31	01.70	108	19.50	04.50	00.44	01.99
Sleep offset weekend (h)	163	05.00	15.00	10.33	01.93	106	06.00	14.55	10.40	02.03
Total sleep on weekday (*)	163	05.00	13.00	08.93	01.57	108	05.00	15.17	08.96	01.97
Total sleep on weekend (*)	162	04.00	15.00	09.97	01.91	105	04.50	16.00	09.83	01.93
Estimated social jetlag*#	160	00.00	06.25	02.20	01.31	104	00.00	06.75	02.52	01.64

NB: HDL, high-density lipoprotein; for descriptive comparison, consider the following mean( ± SD) values in a United States 8-18-year-old sample, for girls and boys, respectively (78): BMI: 22.0(0.2), 21.89(0.1); % body fat: 29.0(0.3), 22.2(0.3); Waist/height (ratio): 0.492(0.003); 0.479(0.002); Triglycerides (mg/dl): 82.8(2.6), 91.6(2.3); Systolic blood pressure (mmHg): 108.3(0.8); 112.1(0.5); Diastolic blood pressure (mmHg): 63.3(0.4); 62.3(0.4); glycated hemoglobin (based on 79): 5.1(0.01), 5.2(0.02).

*Total sleep hours on weekdays and weekend and social jetlag values are the number of hours. For example, minimum value of social jetlag for girls, which is 0.00, means that the difference of sleep midpoint of weekday and weekend was zero hour and maximum was 6.25 hours.

#Social jetlag did not present normal distribution: for girls, 25, 50, and 75% were 1.25, 2.00, and 3.00, respectively. Percentiles 25, 50, and 75% for boys were 1.25, 2.12, and 3.50, respectively.

### 3.2 Cardiometabolic status latent factor

We started with 8 cardiometabolic variables [BMI, percentage of body fat, waist/height ratio, systolic and diastolic blood pressure, glycated hemoglobin, triglycerides, and total cholesterol/HDL (ratio)] to find the single factor structure. The best fitting unidimensional cardiometabolic latent factor was comprised of the following variables (see details of the method to select the indicators in the **Supplementary File 1**): anthropometric measurements (BMI, waist/height ratio), systolic blood pressure and total cholesterol/HDL (ratio). In data of girls, PPP was 0.262, showing adequate fit. The same model for boys converged, but reached a poor fit: PPP equal to 0.041.

**Figure 2:**
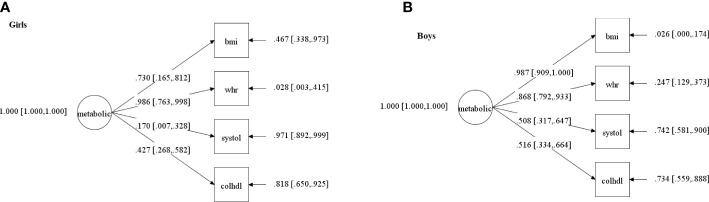
Confirmatory factor Analysis of the cardiometabolic status latent factor in: **(A)** girls; **(B)** boys. NB. The circle indicates the metabolic status latent factor (meta), which represents the common and error-free variance of four observed variables (squares): bmi=body mass index; systole=systolic blood pressure; chol/HDL=total cholesterol/high-density lipoprotein ratio; whr=waist/height ratio. Values on the arrows pointing from the latent factor towards the observed variables are factor loadings (with confidence intervals between square brackets), that is, the extent to which each cardiometabolic variable contributes to the latent score - the higher the loading, the higher the contribution-. Values next to arrows on the right, pointing towards the observed variables, are errors (represent the portion of variance in each cardiometabolic variable that does not share variance with the latent factor). Both models presented acceptable fit indices (described in the text), which is a necessary step to conduct the path analyses that include these latent scores and are described in **Figure 3**.

PPP and 95% confidence interval for the difference between the observed and replicated Chi-square χ2 values of all tested models are shown in **Supplementary File 1**; **Table 2S**. PSR of each of these models are shown in the **Supplementary File 1**; **Table 3S**. The standardized factor loadings and their 95% credible interval of the selected models per sex are shown in **Figure 2**.

### 3.3 Path analyses of the association of SJL with the cardiometabolic latent factor by sex

**Figure 3:**
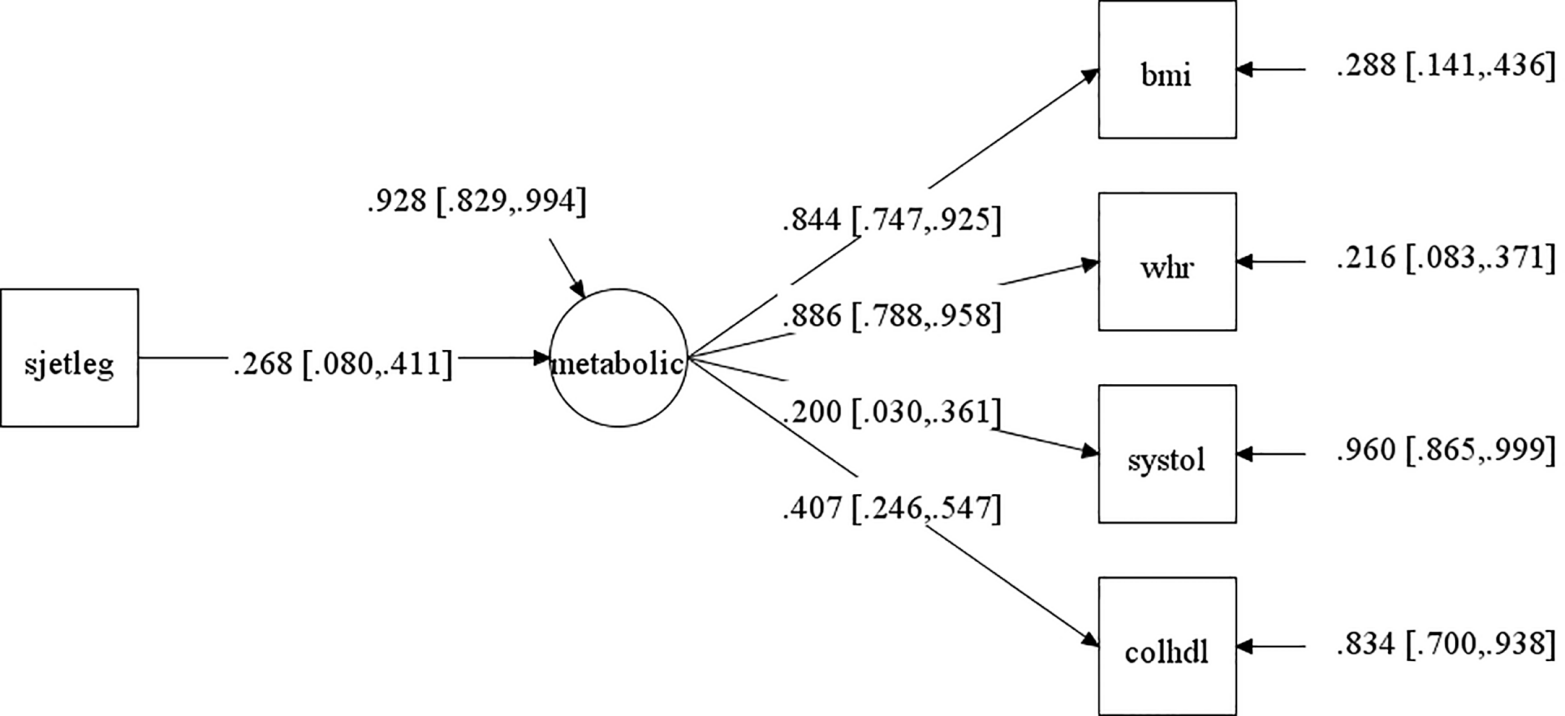
Path analysis of the association of social jetlag (SJL) with the cardiometabolic latent factor (metabolic) for girls without controlling for development (pubertal status or age). NB. This models had acceptable fit indices (described in the text). The arrow pointing from SJL to the circle (which represents the cardiometabolic latent factor) is the main result of interest and represents the correlation between these values. The 95% confidence intervals (CI) of this correlation is represented between square brackets (the range from upper and lower 95% CI must not cross the zero value to be statistically significant, as is the case). The cardiometabolic latent factor (circle) reflects the common variance between the cardiometabolic observed variables (indicate in squares): bmi=body mass index; systole=systolic blood pressure; chol/HDL=total cholesterol/high-density lipoprotein ratio; whr=waist/height ratio. Values on the arrows pointing from the latent factor to observed variables are factor loadings (with confidence intervals between square brackets), that is, the higher the values, the higher the contribution to the latent factor. Values next to arrows on the right, pointing towards the observed variables and toward the cardiometabolic factor, are errors (portion of variance of each variable that does not share variance with the latent factor).

First, we analyzed the association of SJL on the cardiometabolic latent factor, separately by sex, to investigate if our data confirmed this association for adolescents without considering their individual ages and pubertal status. PPP and 95% confidence interval for the difference between the observed and replicated Chi-square χ2 values are shown in the **Supplementary File 1**; **Table 2S**. The convergence criterion are shown in **Supplementary File 1**; **Table 3S**. In girls, the PPP for this model was 0.613, which is a good fit. The standardized factor loadings and their 95% credible interval are shown in **Figure 3**. The standardized regression coefficient was *β* = 0.268, CI [0.080 to 0.411], showing that with every increase of one standard deviation in SJL, the cardiometabolic latent trait rose by 0.268 (note, however, that factor loading of systolic blood pressure was less than 0.4); R squared was 0.072. The comparable model for boys did not converge.

### 3.4 Full path analyses controlled for age or pubertal status on the cardiometabolic latent factor

Next, we controlled for PDS scores in the cardiometabolic latent factor and, in separate models, for age instead of PDS scores only using data of girls because the path model for boys including SJL did not show adequate fit to go forward to run the full path model. The model with data from girls controlled for both PDS and age did not converge (see **Tables 2S**, **3S** in the **Supplementary File 1**).

### 3.5 *Post hoc* exploratory analyses of the association of SJL and cardiometabolic indicators and their change across development

Because the path analyses of data controlled for age and PDS did not fit the data well, we could not determine whether developmental changes exerted an effect on the association of SJL and cardiometabolic status. We therefore carried out additional *post hoc* analyses to explore the extent to which other types of statistical analyses were able to capture whether age and pubertal status had effects on SJL and cardiometabolic indicators, per sex.

Correlations corrected for multiple comparisons between SJL, cardiometabolic and developmental markers for both sexes analyzed separately showed that SJL was higher at older ages and at more mature pubertal stages (**Table 2**). BMI was also higher in older and more sexually matured individuals, but only in girls. Differently, for boys, the only cardiometabolic change across development was a higher systolic blood pressure in older individuals. All these significant associations were small (did not exceed r=0.40). Importantly, correlations of SJL with cardiometabolic indicators did not survive correction for multiple comparisons, which is not usually controlled for in the literature.

**Table 2:** Linear person intercorrelation (r) of social jetlag (SJL), cardiometabolic indicators in the cardiometabolic latent factor and developmental variables (age in months; Pubertal Development Scale scores: PDS), per sex.

		SJL (h)	Body Mass Index (kg/m^2^)	Systolic blood pressure (mmHg)	Total cholesterol/HDL (mg/dl)	Waist/height (ratio)
**Girls (N=164)**	SJL (h)	1	0.226	0.038	0.158	0.206
	PDS	**0.261**	**0.305**	0.089	-0.168	0.061
	Age	**0.229**	**0.293**	-0.007	-0.035	0.016
**Boys (N=105)**	SJL (h)	1	0.200	0.092	-0.083	-0.022
	PDS	**0.384**	0.153	0.213	-0.093	-0.166
	Age	**0.398**	0.196	**0.325**	-0.085	-0.190

NB. values in bold indicate statistically significant correlations after Holm’s correction method; other values were not statistically significant with Holm’s corrections.

Regarding the exploratory MANCOVA with data from girls, the model including SJL and PDS scores had Pillai’s Trace result that showed that there was only a main effect of PDS scores (F_(1,124)_=7.881, p<0.001, η_p_^2 ^= 0.203), while SJL (F_(1,124)_=1.565, p=0.188) and their interaction (F_(1,124)_=2.122, p=0.082) were not significant. Follow-up univariate analyses of between-participant effects showed that this effect was driven by the association of PDS scores with body mass index (BMI) and total cholesterol/HDL, but did not relate to SJL. - For BMI, multivariate result values were F_(1,127)_= 4.887, p=0.029, η_p_^2^ = 0.037 (small effect size); unstandardized regression coefficients (B) was 1.903, indicating that for every unit increase in PDS scores there was an increase of 1.903 in BMI. For total cholesterol/HDL, results were F_(1,127)_= 11.085, p=0.001, η_p_^2^ = 0.080 (medium effect size); the B was -0.485, indicating that for every unit increase in PDS scores there was a decrease of 0.485 in total cholesterol/HDL (that is, less cardiometabolic risk in more sexually mature participants). Adjusted R^2^ of BMI and total cholesterol/HDL were 0.090 and 0.091, respectively, indicating that about 9% of the variance of BMI and cholesterol/HDL was explained by the whole model (small effect sizes). All other factors failed to reach significant effects (p>0.05).

**Figure 4:**
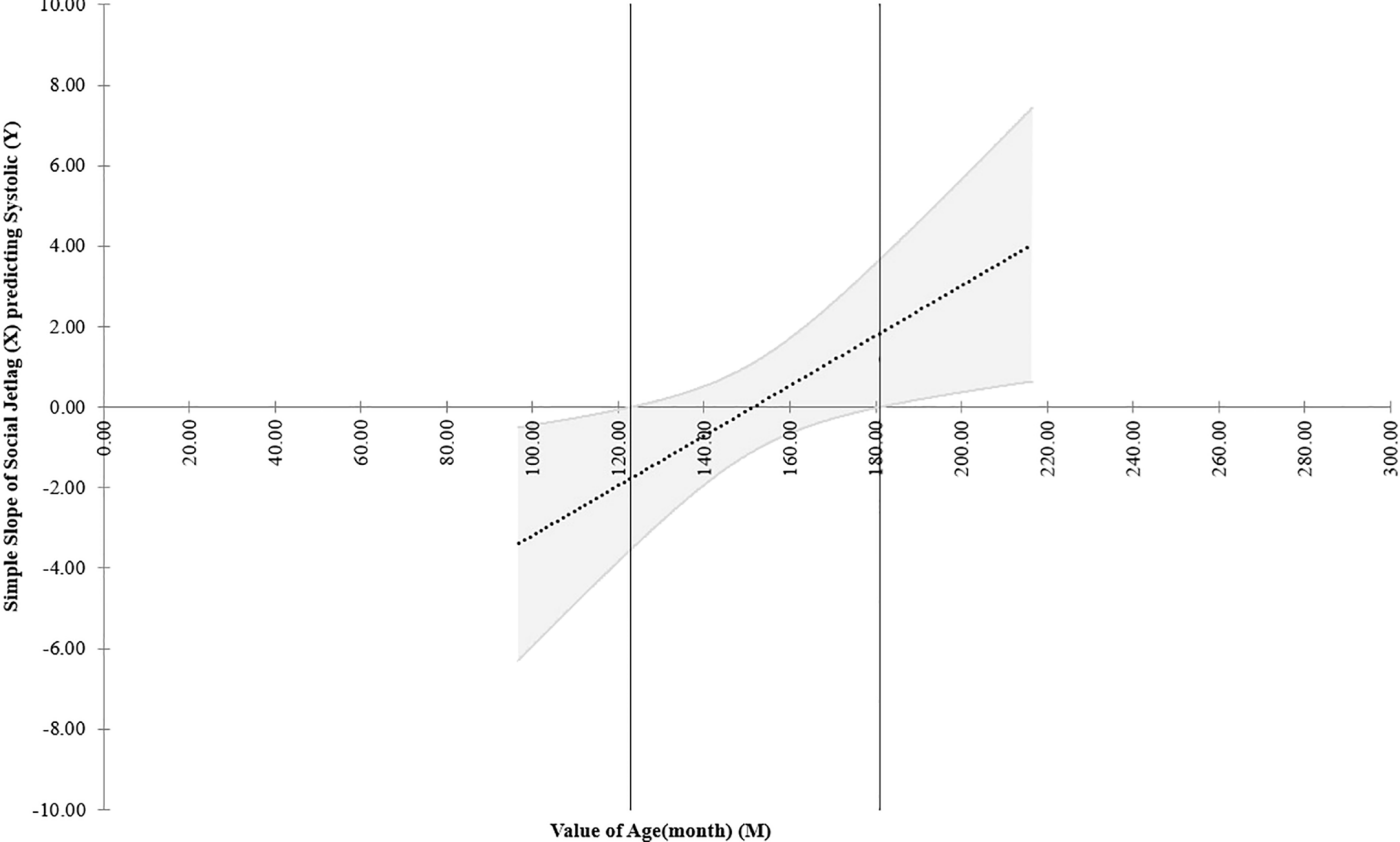
Johnson-Neyman plot of the effect of the interaction of social jetlag and systolic blood pressure according to age (in months) considering data of girls in the *post hoc* multivariate exploratory analyses. NB: The dotted line shows the regression of the interaction of social jetlag and systolic blood pressure across age and the upper and lower parallel lines indicates the ±95% confidence intervals. This Figure shows that the link (interaction) between social jetlag and systolic blood press (y axis) is only significant between below 120 and above 180 months of age, or 10-15 years of age (indicated outside the two vertical lines), whereas in between these aged, which comprise the great majority of the sample, the confidence intervals cross zero on the y axis and the effect is therefore non-significant.

In the equivalent MANCOVA model with data of girls, including age instead of the PDS scores, Pillai’s Trace result showed that there was a main effect of SJL (F_(1,124)_=2.741, p= 0.032, η_p_^2^ = 0.081), age (F_(1,124)_= 6.802, p<0.001, η_p_^2^ = 0.180), and their interaction (F_(1,124)_= 2.948, p=0.023, η_p_^2^ = 0.087). This interaction was driven only by association of age with systolic blood pressure (F_(1,127)_=5.938, p= 0.016, η_p_^2^ = 0.045, small effect size; B=0.067; R^2^ = 0.023). This model explained only 2.3% of the variance of systolic blood pressure so is a negligible effect. Additionally, Johnson-Neyman plots of this interaction (**Figure 4**) showed that it was significant only below around the age of 120 months (10 years of age) and above 180 months (15 years), while in between (which comprises the great majority of our sample) the interaction was not significant because confidence intervals of the regression crossed zero on the y axis in this age interval.

The pattern of exploratory MANCOVA results for boys was different from that of girls, showing no significant associations among the variables, which might explain why the data for this sex did not fit the path analyses models. More specifically, in the model including pubertal development there was no main effect of SJL (F_(1,78)_=1.027, p=0.399), PDS (F_(1,78)_=1.017, p=0.404) or their interaction (F_(1,78)_=1.431, p=0.231). In the model with data of boys using age instead of PDS scores there were also no significant main effect of SJL (F_(1,78)_=1.568, p= 0.191), age (F_(1,78)_=1.940, p= 0.112) or their interaction (F_(1,78)_=1.801, p=0.137).

## 4 Discussion

In this study of a non-clinical sample of early adolescents, we found an average of around a little over two hours of self-reported SJL, which is fairly consistent with other studies that assessed participants of similar ages from other countries (5, 19, 23, 25). However, unlike most studies (8, 18, 41, 43–45), we did not find significant sex differences in this respect, in agreement with Randler et al. (5). In girls, higher SJL was associated with more adverse scores in a continuous cardiometabolic latent factor (shared variance of BMI, waist/height ratio, systolic blood pressure and total cholesterol/HDL ratio), despite the general healthy profile of the sample as a whole in terms of anthropometric/cardiometabolic health (descriptively compared with average data from the United States: 78, 79). This finding extends results on the positive association of SJL and cardiometabolic risk beyond increases in adiposity found in previous studies in adolescents which used other statistical approaches with more limitations (18–23) that will be discussed below. Nonetheless, despite having used a latent factor approach, our path analyses were not very informative regarding the role of pubertal status and age on the association of SJL and cardiometabolic status. Next, we discuss the findings in more detail.

### 4.1 The cardiometabolic latent factor

Our unidimensional cardiometabolic latent factor fitted the data, mirroring results of various previous studies in adolescents (10, 49, 69), adults and elderly (71, 72) from other populations, despite the fact that we used non-adjusted raw scores and a different combination of indicators to form this latent factor (see **Supplementary File 1** for a detailed comparison of our and their latent factors). This is unlikely to be a limitation of our study because we started off with similar biomarkers that were used in these publications, all of which are regarded as related to cardiometabolic status (47). Crucially, a property of latent factors such as those modeled here is that if they are found to have adequate fit indices this means that the latent construct of interest, which was measured indirectly through the shared variance of multiple cardiometabolic biomarkers (also called indicators), actually exists, independently of the indicators used to measure it (46). Therefore, indicators that theoretically reflect the same construct can be used interchangeably across studies and still measure the same construct. Stated differently, our findings show that cardiometabolic risk can be determined as a single latent factor, even in non-clinical samples of adolescents, enabling replications of findings which are not usually possible when different studies use different biomarkers. This has a great advantage because this latent factor can be established even if different cardiometabolic indicators are used across studies. This finding in itself, regardless of the effects of this latent factor on SJL, is a contribution to the literature because it mirrors prior results (10, 49, 69) in a sample from a developing nation with different characteristic from that of other populations of adolescents assessed in the literature. Nonetheless, usually at least three indicators (46) must be used to build a reflective unifactorial latent structure (here we used four).

### 4.2 Associations of SJL and cardiometabolic latent profiles

The association of SJL and cardiometabolic latent risk in girls found here has only previously been consistently shown in respect of increases in adiposity in adolescence (17–23) and was not identified when using a non-latent composite measures of cardiometabolic risk in a large sample of non-clinical early adolescents (21). Although Cespedes Feliciano et al. (21) also assessed cardiometabolic status as a continuous variable (averaging across many standardized indicators), thereby characterizing the participants on a continuum from a better to a worse cardiometabolic profile, differently from their approach our latent factor allowed us to show that the higher the SJL, the worse the shared variance of cardiometabolic measures beyond adiposity. This was clear for females only (without considering age or pubertal status), for which acceptable fit indices were found. In other words, our statistical approach was more sensitive to this effect and picked up this association in girls either because they are more prone to this relation as hypothesized (8, 18, 41, 43–45) or because our sample had more girls. This corroborates findings that components of cardiometabolic risk and adverse endocrine profiles other than adiposity are associated to SJL in pediatric populations (27) and other sleep disturbances (e.g., short sleep duration and bad quality sleep (31–34). The reason for this higher sensitivity of a latent factor is due to the fact that a cardiometabolic latent factor represents the shared variance of different continuous parameters that express an underlying theoretical cardiometabolic construct and partials out the specific effects of individual indicators (46). This makes this analytic approach advantageous to assess its association with SJL compared to: 1) averaging across (21, 35) or combining various indicators in multivariate analyses (27); 2) using a metabolic syndrome index (28, 30, 31, 34), for which there is no consensus in terms of which indicators should be used (49), their cutoff scores or for which adjustments (e.g., for age, sex and pubertal status) should be made (51–54); and 3) using multiple analyses, one for each cardiometabolic-related variables, which still seems to be the norm in the sleep literature. In point of fact, our *post hoc* exploratory correlations of SJL with each cardiometabolic indicator did not survive corrections for multiple comparison in either sex. This is so because these composites or use of many separate correlation analyses per indicator do not account for the fact that cardiometabolic variables are intercorrelated and that carrying out multiple analyses per variable can lead to false positive results, distorting statistical findings. Therefore, determining a cardiometabolic latent factor may better allow future replications of the associations of SJL and cardiometabolic risk compared to the prevailing approaches used so far to establish this relation. Although these effects were found here to be present in healthy girls, this approach may be more important when studying clinical samples of adolescent and may also be more adequate to analyze the effects of sleep disturbances other than SJL in adolescents on cardiometabolic risk. The reason is that the abovementioned statistical inadequacies possibly led to the inconsistency in the individual markers/components of cardiometabolic dysregulation found to be associated with sleep problems (31–34, 80), a relation that has not been found when using non-latent cardiometabolic composites [metabolic syndrome index: (31, 34) the exception being (35)].

Regarding the contribution of each indicator to the latent factor, we stress that BMI and waist/hight ratio made by far the highest contribution in all models, which can explain why SJL has so often been found to relate to adiposity (17–23) (for different results, in possibly underpowered studies, see 24, 26). Although our non-clinical sample had a general healthy cardiometabolic profile, it should be noted that a worse latent cardiometabolic score, even if mostly apparent through a higher BMI, may exert carryover effects that can culminate in the development of the metabolic syndrome (80, 81), which doubles in older compared to younger adolescents (39).

Having obtained an adequate latent cardiometabolic score and finding this to be related with SJL in girls posed the following question: does this latent construct alter SJL or is the inverse true? Although there is no consensus in the literature regarding the mechanisms through which SJL and cardiometabolic risk are interrelated, it has been assumed that disruptions of central and/or peripheral circadian rhythms, beyond the effects of sleep duration/quality, lead to chronic stress and, through this (12–14) and other pathways, dysregulate energy homeostasis (3, 11, 27). Indeed, it is notoriously difficult to differentiate the physiological effects of chronic stress (allostatic load) from those of the metabolic syndrome (15) even at the latent trait level (16). Adolescents who present high SJL have also been found to have worse eating habits, a possible consequences of SJL-induced changes in appetite (22, 82), which potentiates cardiometabolic problems associated with SJL (in animal models: (83). Additionally, a meta-analysis found a positive dose–response relationship of the metabolic syndrome in adults with the duration of exposure to shiftwork (inherently linked to SJL) (81). Prospective studies in pediatric samples also show that short sleep duration, which is often associated with higher SJL, can lead to the development of higher BMI/obesity (26, 84). However, the specific relation between SJL and cardiometabolic markers was not determined in these studies. Hence, it seems that SJL may lead to cardiometabolic changes and not vice-versa, although this was not determined here because we used a cross-sectional design.

### 4.3 Relations of SJL and cardiometabolic latent profiles across sex, age and pubertal status

The full path models controlling for age and PDS scores did not have adequate fit. Follow up exploratory correlation and multivariate analyses showed that cardiometabolism (considering indicators individually with correlations and as a multivariate) was not clearly related to SJL when age and pubertal status were accounted for, so these developmental variables were likely inconsistent with the tested full path models, leading to inadequate fits. In other words, it could be that age/PDS scores may have little to do with the association of SJL with metabolism, the same being true when considering age. The only exception was a very small relation of SJL with systolic blood by age in girls, but this correlation reached a very low effect size and was limited to the extreme ages of our sample. This interaction was not modeled in the full path analyses so it cannot be excluded that it interfered with model fit. Admittedly, the lower sample size considering boys probably influenced this lack of fit.

Regarding the exploratory correlations (corrected for multiple comparisons), for which our sample size can be considered large enough, SJL increased in more mature individuals (older and more sexually developed adolescents), as expected (4, 36). Additionally, BMI was positively related to both chronological and sexual maturity, but only in girls, which also confirms prior findings of higher accumulation of adiposity in this sex (40–43). In the case of boys, the only developmental association with cardiometabolic variables was that of age and systolic blood pressure, which has also been shown to have higher weight in metabolic latent factor in this sex in some ethnicities (10). Despite this, SJL was not significantly correlated with any of the cardiometabolic indicators once corrections for multiple comparisons was carried out, suggesting that some of the reported associations found in prior studies were inflated by type I errors, indicating a questionable link of chronological misalignment (SJL) and worse cardiometabolic profiles *when cardiometabolic indicators are analyses separately* in either sex. Inflated type I errors are avoided using the latent factor approach, which showed here a small positive association of SJL and a latent cardiometabolic risk score, which was only clear in adolescent girls, confirming two studies that found associations of SJL and adiposity only in this sex (18–21).

Prior studies did not investigate the effects of developmental stage on SJL, having usually averaged effects across different ages and/or sexes, or used age/sex adjusted measures in some or all variables. Therefore, although we cannot corroborate our hypotheses of a possible role of chronological and/or pubertal development across the first half of adolescence on the association between SJL and cardiometabolic status, our results show the clear need for further investigations on this issue, especially in clinical samples with either cardiometabolic problems and/or developmental issues. We nonetheless show that age and pubertal status potential influence on the association of SJL and worse cardiometabolic profiles must be analyzed separately in girls and boys as they mature and grow older because this varies widely across sex. Merely statistically controlling for age, pubertal status and/or sex is probably not adequate because these variables likely differently influence SJL and cardiometabolic status, which have not yet been confirmed as invariant (i.e., that they measure the same constructs) across these variables (85).

### 4.4 Conclusion

We conclude that in early adolescence SJL is associated not only with higher adiposity (BMI and waist/height ratio), but also with its shared variance (continuous latent factor) with other indicators of adverse cardiometabolic profiles (higher total cholesterol/HDL ratio and systolic blood pressure) in typically developing girls, but results were not clear in boys.

### 4.5 Practical applications

Our findings showed that a worse cardiometabolic profile was already present in a non-clinical sample of early adolescent girls who had higher SJL. Given the increasing prevalence of metabolic syndrome in adolescents worldwide, our results highlight the need for interventions to help reduce the global burden of cardiometabolic ill health (36), and should serve as a warning that measures must be taken to improve adolescent sleep schedules, particularly for girls who tend to exhibit higher SJL in most studies (8, 18, 41, 43–45). Reducing SJL and improving cardiometabolic health can be attained by: 1) delaying school start times so that adolescents can get more sleep during school nights and therefore decrease the need to compensate for lost sleep on days off (e.g. 86-88); 2) using experimentally successful behavioral strategies in adolescents to extend sleep, including help in organizing schedules to allow earlier bed time, having written instructions and parental support, reducing caffeine intake (89) and, importantly, decreasing electronic screen time at night because this disrupts sleep patterns, particularly in adolescence (88). Improving adolescent eating habits, increasing their levels of physical activity (88) and avoiding exposure to endocrine disrupters (90) are also means of decreasing cardiometabolic risk that is not directly associated with sleep problems. Establishing the mediating and/or moderating contribution of these factors to the association of cardiometabolic risk and SJL were not the aim of the present study, but should be explored in coming experiments. Furthermore, future studies should explore how age and pubertal status contribute to the relation of SJL and cardiometabolic profiles during the pubertal transition, separately in boys and girls, and whether this increases in older adolescence, especially because: 1) children and adolescents seem to be particularly vulnerable to develop obesity due to short sleep duration (26, 84, 91), especially girls (91); 2) rates of metabolic syndrome double in older compared to younger individuals (39); and 3) SJL increases across adolescence (3–5).

### 4.6 Limitations

This study has some limitations that should be noted. First, the cross-sectional design does not enable the determination of whether SJL impairs cardiometabolic status or vice-versa. Second, the path model analyses could have had a better fit if we had tested a larger sample, especially a larger sample of boys, who were under represented compared to girls and likely led to a poor but convergent cardiometabolic latent factor. Third, the SJL and pubertal status measures were obtained here through self-reports, which only partially correspondent to objective measures of these variables (20, 21, 37). For instance, lower SJL (of around one hour) has been found in studies that used actigraphy compared to self-report (20, 21). However, although the presence of SJL can be determined objectively using actigraphy, this is usually only assessed over a limited period of time. Therefore, these results might not represent usual disrupted sleep-wake patterns evaluated with a subjective rating about general sleep habits, as was done in the current study (29), which may be more highly associated, in the long-term, with cardiometabolic dysregulation. Furthermore, there are cultural differences in sleep timing (29), which might explain why Tamura et al. (8) reported only one hour of self-reported SJL, while here, and in other publications (5, 19, 25), SJL was closer to two hours. Lastly, our statistical models did not control for sleep durations, sleep quality, nutritional status or physical activity, which have been shown to be related with the association of SJL and adiposity (18, 21, 26, 90), nor for exposure to endocrine disrupters which affect metabolism and pubertal timing (90). We also took the decision not to control for ethnicity to avoid fallacious racial stereotypes, despite suggestions that race influences cardiometabolic profiles (48) and pubertal status (37). The reasons were that Brazil has a highly admixed population with unclear skin color boundaries (92) and that race labels have a much lower association with genetic ancestry than most of the literature recognizes (93, 94).

## Data availability statement

The original contributions presented in the study are included in the article/**Supplementary material** and available at https://osf.io/5mv6w. Further inquiries can be directed to the corresponding author.

## Ethics statement

This study received ethical approval from the Comitê de Ética em Pesquisa of the Universidade Federal de São Paulo (CAAE 56284216.7.0000.5505). Informed assent from participants was obtained and parents/legal guardians provided writen informed consent.

## Author contributions

Conception, design, supervision of the study and data collection: SPo and HC-M. Analysis: SPa and HC-M. Interpretation of the data: all authors. Drafting of the manuscript: SPo. Critical contributions to the draft and approval of the final version: all authors. Public responsibility for the content of the article: SPo. All authors contributed to the article and approved the submitted version.

## Footnotes

1 The metabolic syndrome index is classically determined by summing points assigned for the presence of a series of metabolic risk components based on arbitrary cut-off score values (e.g., obese versus non obese, hypertensive versus normotensive); when these points exceed a certain (also arbitrary) number, participants are classified as presenting the syndrome (49). The problem of using a metabolic syndrome risk measures is that there is no consensus on which variables (indicators) should be used to determine this syndrome, nor the ideal cut-offs to establish adolescents as having metabolic syndrome. Up to 40 different unique definition of this syndrome are found in the literature (49, 50), which differently try to account for developmental trends in factors such as age, sex, ethnicity or pubertal status (51–54) that are not comparable. With artificial splits such as those proposed to determine the presence of metabolic syndrome, individuals close to, but on opposite sides of the cut-off value can actually be more similar than others within each artificially determined group, which causes various problems when interpreting data (55). For instance, (51) classified a large sample of youngsters of an overweight sample using 8 different criteria and found that the prevalence of this syndrome varied from 6 to 39%, with only 2% of the sample having been classified as presenting the syndrome in all criteria. Another drawback in using metabolic syndrome indexes is that summing points of individual components does not account for the correlations among them and/or possible differential influences (or weights) of each component in characterizing metabolic problems (10).
