# Supplementary material for: Social jetlag is associated with adverse cardiometabolic latent traits in early adolescence: an observational study
Source: Front Endocrinol (Lausanne). 2023 Jul 4;14:1085302. doi: 10.3389/fendo.2023.1085302 (PMC10352840; doi:10.3389/fendo.2023.1085302)
Supplement: Supplementary file 2 [file DataSheet_2.pdf]

Supplementary File 1

This Supplementary file included details regarding the Methods, Results, and Discussion sections of the main document. Supplementary file 2 contains the Mplus syntax.

METHODS

Determining the metabolic latent factor

Because there is no consensus of which indicators should be used to build a cardiometabolic latent score, we followed a series of steps in the interest of transparency. Firstly, because we had no prior data to base our to decide which metabolic indicators should compose the latent factors of interest here, we considered variables of adiposity and cardiometabolic risk (Sung & Rhee, 2007; Barbosa-Leiker, 2013; Shi et al. 2015; Weihe & Weihrauch-Blüher, 2019) similar to those used by Ford & Li (2007), Martínez-Vizcaino et al. (2011) and Gurka et al. (2012). However, because the models used in these studies differed and were not consistent, we sought to build a model using all our data with 8 variable [glycated hemoglobin, which indicates the control of body glucose over the last few months; lipid profile, including cholesterol ratio (total cholesterol divided by HDL: Chu et al., 2019), anthropometric/adiposity measurements (BMI, waist/height ratio, percentage body fat) and systolic and diastolic blood pressure]. As mentioned, changing indicators should not affect the latent factor as long as they have all been shown to relate to metabolic syndrome, as was the case (Brown, 2015). Although it is unclear which is the best way to model the cardiometabolic syndrome, we decided to follow the principle of parsimony in assuming it would compose a single factor, as found before (e.g., Düzel et al., 2018; Gurka et al., 2012; Li & Ford, 2007; Pladevall et al., 2006). We did not explore alternative factor structures, although some have also been proposed (see Ford & Li, 2008; Barbosa-Leiker, 2013).

Next, we built Pearson correlation matrixes including all 8 studied cardiometabolic variables to determine how they intercorrelated with each other because indicators have to associate to form reflective latent factors. This was done considering sex separately for girls and boys. . Sex-specific latent factors were obtained because some studies have shown that it is important to consider sex-specific metabolic risk latent factors in adolescents (e.g., Gurka et al., 2012; Li & Ford, 2007), which makes sense considering the different developmental metabolic trajectories in boys and girls and that metabolic sex differences are also present in adults (e.g., Düzel et al., 2018).

The following step was to choose intercorrelations of metabolic indicators higher than [±]0.3 (medium effect size: see Ellis, 2010, page 41) as a minimum cutoff value for our selection of variables to form the latent factor, without correcting for multiple comparisons for the whole sample and separately by sex. To establish the maximum acceptable correlation we used another approach, to avoid high collinearity (Johnston et al., 2018) which could prioritize the selection of variables that are essentially measuring a specific cardiometabolic aspect, potentially biasing the interpretability of the latent factor as a more general cardiometabolic profile marker. Although there is no consensus on how to determine this from correlation matrices, we opted to excluded variables when intercorrelations were $r>0.90$ (Franke, 2010; Kim, 2019).

We then used Mplus to conduce Confirmatory Factor Analyses (CFA) to determine the adequacy of the latent factors, that is, to confirm whether the proposed

theoretical metabolic status model explained the covariance among the observed variables (Schmitt, 2011). If the models did not converge or had unacceptable fit, we removed variables with lower factor loadings until a convergent model was obtained.

RESULTS

Establishing the metabolic latent factor

Correlations matrixes within the metabolic markers for the whole sample, which was used to select the indicators to compose the metabolic latent factors, can be found in Table 1S, together with the same correlations per sex.

Table 1S. Linear Pearson intercorrelation (r) among metabolic status variables.

	**Metabolic status variables**	**A**	**B**	**C**	**D**	**E**	**F**	**G**	**H**
**Girls**	A - Body mass index (kg/m^2)	1							
	B - Body fat (%)	**0.912**	1						
	C - Waist/height (ratio)	**0.756**	**0.724**	1					
	D - Systolic blood pressure (mmHg)	0.192	0.147	0.67	1				
	E - Diastolic blood pressure (mmHg)	0.146	0.103	0.137	**0.678**	1			
	F - Triglycerides (mg/dl)	**0.326**	**0.270**	**0.327**	0.074	-0.026	1		
	G - Total cholesterol/HDL (ratio)	**0.332**	**0.263**	**0.306**	0.118	0.030	**0.554**	1	
	H - Glycated hemoglobin (%)	-0.158	-0.188	-0.182	0.030	-0.016	0.056	0.018	1
**Boys**	A - Body mass index (kg/m^2)	1							
	B - Body fat (%)	**0.908**	1						
	C - Waist/height (ratio)	**0.854**	**0.873**	1					
	D - Systolic blood pressure (mmHg)	**0.530**	**0.392**	**0.312**	1				
	E - Diastolic blood pressure (mmHg)	**0.310**	**0.363**	0.253	**0.657**	1			
	F - Triglycerides (mg/dl)	**0.459**	**0.522**	**0.529**	0.253	0.143	1		
	G - Total cholesterol/HDL (ratio)	**0.503**	**0.433**	**0.538**	0.189	0.189	**0.540**	1	
	H - Glycated hemoglobin (%)	-0.064	-0.171	0.007	-0.047	-0.074	0.065	0.145	1
	G - Total cholesterol/HDL (ratio)	**0.405**	**0.214**	**0.467**	0.154	0.093	**0.551**	1	
	H - Glycated hemoglobin (%)	-0.120	-0.171	-0.097	0.017	-0.020	0.046	0.062	1

Note. Correlations above 0.30 our below -0.30 considering the whole sample were used to select variables to compose the first tested metabolic latent factor. All these correlations were significant (bold) even after applying Holm's correction (Holm 1979) method for multiple comparisons; Underlined values did not survive Holm's correction; other values were not statistically significant with and without Holm's corrections.

Glycated haemoglobin was not included in our first tested latent factor because its correlations with the other indicators were very low (rs<±0.19). BMI and % body fat showed multicollinearity in boys and girls (r>0.90) as found before (Pietrobelli et al., 1998), so BMI was prioritized as it is easier to measure in other studies that might aim to replicate our results. The first tested model thus initially included BMI, waist/height ratio, triglycerides, and diastolic and systolic blood pressure, and total cholesterol/HDL, all of which had intercorrelations higher than 0.3 with at least one other variable.

The sex-specific models with all indicators did not have acceptable fit so we first removed diastolic blood pressure because it had the lowest factor loading. These models also did not have acceptable fit. The next lowest indicator was systolic blood pressure but

we opted not to remove it next in order to keep a cardiometabolic indicator in our latent factor. Instead, we removed the next lowest indicator, triglycerides.. These models for both sexes, without diastolic and triglycerides, had acceptable fit and was larger than that including triglycerides instead of cholesterol/HDL. Hence, the best fitting unidimensional metabolic latent factor was comprised of the following variables: anthropometric measurements (BMI, waist/height ratio), systolic blood pressure, and total cholesterol/HDL and these were used to undertake the analyses described in main text where fit indices can be found: to determine the effects of social jetlag (SJL) on the metabolic latent factor per sex, and then this same model controlled for age (or Pubertal Development Scale -PDS- scores) on the outcome, which we named "full model".

Details regarding the other non-fitting models can be found in the Supplementary File 2 - MPlus syntax/code. Table 2S includes all Posterior predictive *p* values (PPP) and 95% confidence interval (CI) for the difference between the observed and replicated Chi-square $\chi 2$ values of the Confirmatory Factor Analyses tested to model cardiometabolism. Table 3S includes Potential Scale Reduction (PSR) and parameters with the highest PSR of the different tested models. These details regarding the path analyses in girls can also be found in these Tables 2S and 3S.

Table 2S: Posterior predictive *p* values (PPP) and 95% confidence interval (CI) for the difference between the observed and replicated Chi-square $\chi 2$ values of the Confirmatory Factor Analyses tested to model cardiometabolism as a latent factor and for the Path Analyses of the association of this latent factor with social jetlag (SJL), and also adjusting for age and Pubertal Development Scale scores (PDS).

				PPP	95% CI	
**Confirmatory factor analysis (CFA)**		**Girls**	Model one	<0.001	97.791	144.319
			Model two	<0.001	14.858	49.427
			Model three	0.182	-13.363	85.290
			Model four	0.262	-16.761	105.958
		**Boys**	Model one	<0.001	56.284	98.069
			Model two	<0.001	21.585	61.894
			Model three	<0.001	6.766	34.954
			Model four	0.041	-2.485	31.357
**Path Analysis**	**SJL**	**Girls**		0.613	-13.023	8.651
		**Boys**		<0.001	12.531	44.020
	**SJL & age**	**Girls**		0.022	1.464	35.865
		**Boys**		<0.001	58.673	94.146
	**SJL & PDS**	**Girls**		0.003	8.458	42.173
		**Boys**		<0.001	37.223	69.591

NB: CFA model one variables: BMI, Waist-Hight ratio (WHr), systolic blood pressure and diastolic blood pressure, triglycerides, and total cholesterol/HDL ratio (Chol/HDL). Model two: BMI, WHr, systolic blood pressure, triglycerides, and Chol/HDL. Model three: BMI, WHr, systolic blood pressure and triglycerides. Model four: BMI, WHr, systolic blood pressure and Chol/HDL, which was the model used for the path analyses. Convergence criterion and proportional scale reduction (PSR) of models 3 and 4 and of the path analyses are shown in Table 3S.

Table 3S: Potential Scale Reduction (PSR) and parameters with the highest PSR of different Confirmatory Factor Analyses (CFA) for both sexes and the Path Analyses of association with social jetlag (SJL) with data of girls.

	Confirmatory Factor Analysis						Path analysis	
	Girls				Boys		Girls	
	Model three		Model four		Model four		Association with SJL	
ITERATION	PSR	HIGHEST PSR	PSR	HIGHEST PSR	PSR	HIGHEST PSR	PSR	HIGHEST PSR
100	1.24	5	2.777	7	3.281	5	2.809	5
200	1.078	12	4.328	5	7.523	5	1.728	13
300			1.423	7	6.538	12	1.198	8
400			1.075	7	6.406	12	1.089	13
500					6.432	12	1.139	8
600					6.279	12	1.036	8
700					3.691	12		
800					1.861	12		
900					1.387	12		
1000					1.206	12		
1100					1.11	1		
1200					1.142	1		
1300					1.176	1		
1400					1.136	8		
1500					1.123	8		
1600					1.086	8		
1700					1.07	1		

NB. Model three: BMI, WHr, systolic blood pressure and triglycerides. Model four: BMI, WHr, systolic blood pressure and Chol/HDL, which was the model used for the path analyses.

DISCUSSION

Comparison of the metabolic latent factor with that of prior studies in pediatric populations

At the age of interest here, three studies modeled cardiometabolism as a single latent factor (unifactorial) and included four or five indicators which varied from study to study but that, in general, coincided with ours. Li & Ford's (2007) latent factor included triglycerides, systolic blood pressure, insulin resistance, and waist circumference, Gurka et al. (2012) considered the same first two components, plus HDL, fasting glucose and

BMI, while and Martínez-Vizcaino et al.'s (2011) model included fasting insulin, waist circumference (like Li & Ford, 2007), triglycerides divided by HDL cholesterol, and mean arterial blood pressure. Li & Ford (2007) and Martínez-Vizcaino et al. (2011) used age- and sex-adjusted scores for all indicators. Differently, Gurka et al. (2012) used age- and sex-specific corrected BMI scores, while these adjustments were not made for the other components, . These developmental adjustments make it impossible to determine how the latent factor changed across ages/pubertal status and sexes. Standardizing all or part of the scores would not allow us to determine the effects of development on social jetlag, cardiometabolism, or their associations, so we used raw scores for all variables. Overall, because all these models and ours converged and had acceptable fit this confirms that the non directly observable (latent) cardiometabolic construct can be gauged as the common variance among a series of cardiometabolic markers.

From our initial set of 8 indicators, four were excluded to build the latent factor (glycated hemoglobin, body fat percentage, diastolic blood pressure and triglycerides). Concerning glycated hemoglobin, it was excluded because it failed to associate with the other variables. In this respect we were not alone. Gurka et al. (2012) also found that fasting glucose had a low factor loading, which they justified as due to the glucose narrow maintenance range expected at this age in healthy youngsters. Body fat was not considered because of multicollinearity with BMI, a necessary precaution suggested by Gurka et al. (2012). Regarding hypertension markers, systolic blood pressure was more related to the other metabolic variables than diastolic blood pressure confirming the former to be a more sensitive metabolic biomarker than other blood pressure indicators [diastolic blood pressure (Li & Ford (2007) and mean arterial blood pressure (Gurka et al. (2012)], although as fond here it tends to have lower factor loading than other metabolic indicators (see Li & Ford, 2007; Martínez-Vizcaino et al., 2011). Triglycerides, which was also excluded, was considered in the metabolic latent factor of Gurka et al. (2012) after log transformation or as triglycerides–to–HDL cholesterol ratio in Li & Ford (2007). It follows that this variable could have had higher factor loading here had we explored other ways of using this variable.

REFERENCES

Barbosa-Leiker C. Factor analysis applied to the metabolic syndrome: A review. In: Berhardt LV, editor. Advances in medicine and biology. volume 59. New York: Nova Science Publishers (2013). 256 p.

Brown TA. Confirmatory factor analysis for applied research. 2nd ed. New York: Guilford Press (2015).

Chu SY, Jung JH, Park MJ, Kim SH. Risk assessment of metabolic syndrome in adolescents using the triglyceride/high-density lipoprotein cholesterol ratio and the total cholesterol/high-density lipoprotein cholesterol ratio. Ann Pediatr Endocrinol Metabol (2019) 24(1): 41-48. doi:10.6065/apem.2019.24.1.41

Düzel S, Buchmann N, Drewelies J, Gerstorf D, Lindenberger U, Steinhagen-Thiessen E, Norman K, Demuth I. Validation of a single factor representing the indicators of metabolic syndrome as a continuous measure of metabolic load and its association with health and cognitive function. PLoS One (2018) 13(12):e0208231. doi:10.1371/journal.pone.0208231

Ellis PD. The essential guide to effect sizes: Statistical power, meta-analysis, and the interpretation of research results. Cambridge Uni Press (2010) doi:10.1017/CBO9780511761676

Ford ES, Li C. Defining the metabolic syndrome in children and adolescents: will the real definition please stand up? J Pediatr (2008) 152(2): 160-164. doi:10.1016/j.jpeds.2007.07.056

Franke GR. Multicollinearity. Wiley international encyclopedia of marketing (2010). https://doi.org/10.1002/9781444316568.wiem02066.

Gurka MJ, Ice CL, Sun SS, DeBoer MD. A confirmatory factor analysis of the metabolic syndrome in adolescents: an examination of sex and racial/ethnic differences. Cardiovasc (2012) 11(1): 1-10. doi:10.1186/1475-2840-11-128

Holm S.. A simple sequentially rejective multiple test procedure. Scand Stat Theory Appl. (1979) 6(2):65–70.

Johnston R, Jones K, Manley D. Confounding and collinearity in regression analysis: a cautionary tale and an alternative procedure, illustrated by studies of British voting behaviour. Quality & quantity (2018) 52(4):1957-76. doi:10.1007/s11135-017-0584-6

Kim JH. Multicollinearity and misleading statistical results. *Korean journal of anesthesiology (2019)*, *72*(6):558-569. https://doi.org/10.4097/kja.19087

Martínez-Vizcaino V, Ortega FB, Solera-Martínez M, Ruiz JR, Labayen I, Eensoo D, Harro J, Loit HM, Veidebaum T, Sjöström M. Stability of the factorial structure of metabolic syndrome from childhood to adolescence: a 6-year follow-up study. Cardiovasc (2011) 10(1):1-8. doi:10.1186/1475-2840-10-81

Pietrobelli A, Faith MS, Allison DB, Gallagher D, Chiumello G, Heymsfield SB. Body mass index as a measure of adiposity among children and adolescents: a validation study. The Journal of pediatrics. 1998 Feb 1;132(2):204-10. doi:10.1016/S0022-3476(98)70433-0

Pladevall M, Singal B, Williams LK, Brotons C, Guyer H, Sadurni J, et al. A single factor underlies the metabolic syndrome: a confirmatory factor analysis. Diabetes Care (2006) 29(1): 113-122. doi:10.2337/diacare.29.1.113

Schmitt TA. Current methodological considerations in exploratory and confirmatory factor analysis. J Psychoeduc Assess (2011) 29(4): 304-321. doi:10.1177/0734282911406653

Shi P, Goodson JM, Hartman ML, Hasturk H, Yaskell T, Vargas J, Cugini M, Barake R, Alsmadi O, Al-Mutawa S, Ariga J. Continuous metabolic syndrome scores for children using salivary biomarkers. PloS one (2015) 10(9):e0138979. doi:10.1371/journal.pone.0138979

Sung KC, Rhee EJ. Glycated haemoglobin as a predictor for metabolic syndrome in non-diabetic Korean adults. Diabet Med (2007) 24(8): 848-854. doi:10.1111/j.1464-5491.2007.02146.x

Weihe P, Weihrauch-Blueher S. Metabolic syndrome in children and adolescents: diagnostic criteria, therapeutic options and perspectives. Curr Obes Rep (2019) 8(4): 472-479.

doi:10.1007/s13679-019-00357-x
