# Supplementary material for: Social jetlag is associated with adverse cardiometabolic latent traits in early adolescence: an observational study
Source: Front Endocrinol (Lausanne). 2023 Jul 4;14:1085302. doi: 10.3389/fendo.2023.1085302 (PMC10352840; doi:10.3389/fendo.2023.1085302)
Supplement: Supplementary file 3 [file DataSheet_3.pdf]

Supplementary File 2 (Mplus code);

This Supplementary file included the Mplus code for all structural equation models.

1. Confirmatory factor analysis for girls and boys, including four models per sex.

```
Title: metabolic_CFA_Girls_model_one;

Data: file is data.txt; !the data available in supplementary material is in .xlsx format and should be changed to .txt and name of variables should be removed to run the code!

Variable: names are ID sex agem BMI FATM WHr Systol Diastol
Trigly ColHDL HbA1c PDS_S sjetlag gobedweekend
wupwend totalsleepend gobedwdays wupwkday totalsleepday;

useobservation Sex EQ 1; !1 is female!
usevariable are BMI WHr Systol Diastol Trigly ColHDL;
Missing are all (-999);

analysis:
estimator= bayes;
ALGORITHM = INTEGRATION;
model:
metabolic by BMI WHr Systol Diastol Trigly ColHDL;

output:
standardized TECH8;
Plot:
type= plot2;
```

```
Title: metabolic_CFA_Girls_model_two;
Data: file is data.txt;
```

Variable: names are ID sex agem BMI FATM WHr Systol Diastol

Trigly ColHDL HbA1c PDS_S sjetlag gobedweekend

wupwend totalsleepend gobedwdays wupwkday totalsleepday;

useobservation Sex EQ 1; !1 is female!

usevariable are BMI WHr Systol Trigly ColHDL;

Missing are all (-999);

analysis:

estimator= bayes;

ALGORITHM = INTEGRATION;

model:

metabolic by BMI WHr Systol Trigly ColHDL;

output:

standardized TECH8;

Plot:

type= plot2;

Title: metabolic_CFA_Girls_model_three;

Data: file is data.txt;

Variable: names are ID sex agem BMI FATM WHr Systol Diastol

Trigly ColHDL HbA1c PDS_S sjetlag gobedweekend

wupwend totalsleepend gobedwdays wupwkday totalsleepday;

useobservation Sex EQ 1; !1 is female!

usevariable are BMI WHr Systol Trigly;

Missing are all (-999);

analysis:

estimator= bayes;

ALGORITHM = INTEGRATION;

model:

metabolic by BMI WHr Systol Trigly;

output:

standardized TECH8;

Plot:

type= plot2;

Title: metabolic_CFA_Girls_model_four;

Data: file is data.txt;

Variable: names are ID sex agem BMI FATM WHr Systol Diastol

Trigly ColHDL HbA1c PDS_S sjetlag gobedweekend

wupwend totalsleepend gobedwdays wupwkday totalsleepday;

useobservation Sex EQ 1; !1 is female!

usevariable are BMI WHr Systol ColHDL;

Missing are all (-999);

analysis:

estimator= bayes;

ALGORITHM = INTEGRATION;

model:

metabolic by BMI WHr Systol ColHDL;

output:

standardized TECH8;

Plot:

type= plot2;

Title: metabolic_CFA_Boys_model_one;

Data: file is data.txt;

Variable: names are ID sex agem BMI FATM WHr Systol Diastol

Trigly ColHDL HbA1c PDS_S sjetlag gobedweekend

wupwend totalsleepend gobedwdays wupwkday totalsleepday;

useobservation Sex EQ 2; !2 is male!

usevariable are BMI WHr Systol Diastol

Trigly ColHDL;

Missing are all (-999);

analysis:

estimator= bayes;

ALGORITHM = INTEGRATION;

model:

metabolic by BMI WHr Systol Diastol

Trigly ColHDL;

output:

standardized TECH8;

Plot:

type= plot2;

Title: metabolic_CFA_Boys_model_two;

Data: file is data.txt;

Variable: names are ID sex agem BMI FATM WHr Systol Diastol

Trigly ColHDL HbA1c PDS_S sjetlag gobedweekend

wupwend totalsleepend gobedwdays wupwkday totalsleepday;

useobservation Sex EQ 2; !2 is male!

usevariable are BMI WHr Systol

Trigly ColHDL;

Missing are all (-999);

analysis:

estimator= bayes;

ALGORITHM = INTEGRATION;

model:

metabolic by BMI WHr Systol

Trigly ColHDL;

output:

standardized TECH8;

Plot:

type= plot2;

Title: metabolic_CFA_Boys_model_three;

Data: file is data.txt;

Variable: names are ID sex agem BMI FATM WHr Systol Diastol

Trigly ColHDL HbA1c PDS_S sjetlag gobedweekend

wupwend totalsleepend gobedwdays wupwkday totalsleepday;

useobservation Sex EQ 2; !2 is male!

usevariable are BMI WHr Systol Trigly;

Missing are all (-999);

analysis:

estimator= bayes;

ALGORITHM = INTEGRATION;

model:

metabolic by BMI WHr Systol Trigly;

output:

standardized TECH8;

Plot:

type= plot2;

Title: metabolic_CFA_Boys_model_four;

Data: file is data.txt;

Variable: names are ID sex agem BMI FATM WHr Systol Diastol

Trigly ColHDL HbA1c PDS_S sjetlag gobedweekend

wupwend totalsleepend gobedwdays wupwkday totalsleepday;

useobservation Sex EQ 2; !2 is male!

usevariable are BMI WHr Systol

ColHDL;

Missing are all (-999);

analysis:

estimator= bayes;

ALGORITHM = INTEGRATION;

model:

metabolic by BMI WHr Systol ColHDL;

output:

standardized TECH8;

Plot:

type= plot2;

----

2. Path analysis, investigating the association of social jetlag and metabolic latent factor, using the CFA model four, per sex.

Title: metabolic_Path analysis_Girls_association of social jetlag and metabolic latent factor;

Data: file is data.txt;

Variable: names are ID sex agem BMI FATM WHr Systol Diastol

Trigly ColHDL HbA1c PDS_S sjetlag gobedweekend

wupwend totalsleepend gobedwdays wupwkday totalsleepday;

useobservation Sex EQ 1; !1 is female!

usevariable are BMI WHr Systol ColHDL sjetleg;

Missing are all (-999);

analysis:

estimator= bayes;

ALGORITHM = INTEGRATION;

model:

metabolic by BMI WHr Systol ColHDL;

metabolic on sjetleg;

output:

standardized TECH8;

Plot:

type= plot2;

Title: metabolic_Path analysis_Boys_association of social jetlag and metabolic latent factor;

Data: file is data.txt;

Variable: names are ID sex agem BMI FATM WHr Systol Diastol

Trigly ColHDL HbA1c PDS_S sjetlag gobedweekend

wupwend totalsleepend gobedwdays wupwkday totalsleepday;

useobservation Sex EQ 2; !2 is female!

usevariable are BMI WHr Systol ColHDL sjetleg;

Missing are all (-999);

analysis:

estimator= bayes;

ALGORITHM = INTEGRATION;

model:

metabolic by BMI WHr Systol ColHDL;

metabolic on sjetleg;

output:

standardized TECH8;

Plot:

type= plot2;

---

3. Full Path analysis, investigating the association of social jetlag and metabolic latent factor, controlling the effect of puberty developmental score and age, using the CFA model four, for girls.

Title: metabolic_Full path model_girls;

Data: file is data.txt;

Variable: names are ID sex agem BMI FATM WHr Systol Diastol

Trigly ColHDL HbA1c PDS_S sjetlag gobedweekend

wupwend totalsleepend gobedwdays wupwkday totalsleepday;

useobservation Sex EQ 1; !1 is female!

usevariable are agem BMI WHr Systol ColHDL sjetleg;

Missing are all (-999);

analysis:

estimator= bayes;

ALGORITHM = INTEGRATION;

model:

metabolic by BMI WHr Systol ColHDL;

metabolic on sjetleg agem;

output:

standardized TECH8;

Plot:

type= plot2;

Title: metabolic_Full path model_girls;

Data: file is data.txt;

Variable: names are ID sex agem BMI FATM WHr Systol Diastol

Trigly ColHDL HbA1c PDS_S sjetlag gobedweekend

wupwend totalsleepend gobedwdays wupwkday totalsleepday;

useobservation Sex EQ 1; !1 is female!

usevariable are agem BMI WHr Systol ColHDL sjetleg;

Missing are all (-999);

analysis:

estimator= bayes;

ALGORITHM = INTEGRATION;

model:

metabolic by BMI WHr Systol ColHDL;

metabolic on sjetleg agem;

output:

standardized TECH8;

Plot:

type= plot2;
